# Covid-19 overlapping with systemic sclerosis

**DOI:** 10.1590/0037-8682-0450-2020

**Published:** 2020-09-21

**Authors:** Rachel Zerbini Mariano, Ana Paula Toledo del Rio, Fabiano Reis

**Affiliations:** 1 Universidade Estadual de Campinas, Faculdade de Ciências Médicas, Departamento de Radiologia, Campinas, SP, Brasil.; 2 Universidade Estadual de Campinas, Faculdade de Ciências Médicas, Departamento de Medicina Interna, Campinas, SP, Brasil.

**FIGURE 1:**
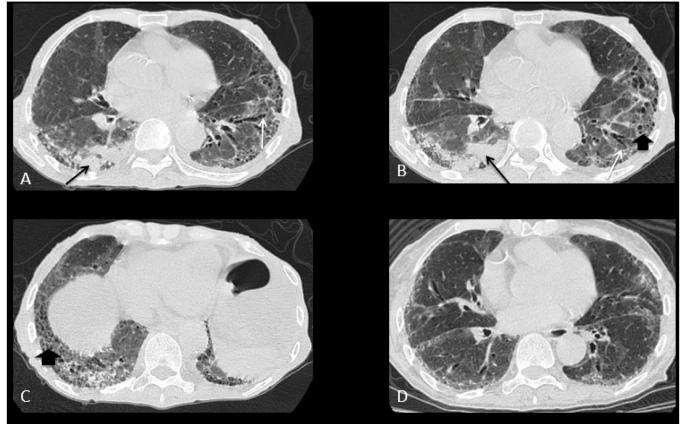
(A) and (B): Chest CT, pulmonary window, axial: right lower lobe consolidation (black arrows); (A), (B), and (C): Findings consistent with UIP: predominantly peripheral and basal interlobular septal thickening, reticulations, traction bronchiectasis (white arrows), and honeycombing (thick arrow); (D): CT performed eight months prior, without consolidation.

A 73-year-old woman with systemic sclerosis (SSc) sine scleroderma presented with Raynaud's phenomenon, esophageal motor disorder with significant dilation, and lung involvement characterized by usual interstitial pneumonia (UIP). Nailfold capillaroscopy revealed a scleroderma pattern. She had antinucleolar autoantibody. The patient was dehydrated, malnourished, admitted to the emergency department, and not taking any immunosuppressants at admission. Real-time polymerase chain demonstrated SARS-CoV-2 infection. A chest computed tomography (CT) revealed right lower lobe consolidation and findings consistent with UIP-predominantly peripheral and basal interlobular septal thickening, reticulations, traction bronchiectasis, and honeycombing (Figure 1A-C). A CT scan eight months prior showed no consolidation (Figure 1D). 

In interstitial lung disease associated with SSc, the most common initiator is injury to alveolar epithelial and vascular endothelial cells; inflammatory pathways activate profibrotic stimuli that produce varying degrees of inflammation and fibrosis. The main interstitial patterns observed are nonspecific interstitial pneumonia and UIP, patterns of which may share some features with Covid-19 pneumonia, such as ground-glass opacities, reticulation, and subpleural lines^1^ ^,^ ^2^. 

CT findings of Covid-19 mainly include, subpleural and predominantly peripheral ground-glass opacities, a crazy-paving pattern, and/or consolidation with air bronchograms, usually with bilateral and multilobar involvement^3^. However, when a patient presents with a pre-existing pathology (such as UIP) comparison with previous CT findings should be done to avoid missing a diagnosis of pulmonary involvement caused by SARS-CoV-2 infection. The presence of interstitial lung disease and ongoing immunosuppressive treatment may place patients with SSc at risk of developing more severe disease and higher mortality when infected by SARS-CoV-2^2^. 
